# Disability progression among leprosy patients released from treatment: a survival analysis

**DOI:** 10.1186/s40249-020-00669-4

**Published:** 2020-05-24

**Authors:** Aleksandra Rosendo dos Santos, Pãmela Rodrigues de Souza Silva, Peter Steinmann, Eliane Ignotti

**Affiliations:** 1 Health Sciences Graduation Program, Federal University of Mato Grosso (UFMT), Cuiabá, Brazil; 2 Faculty of Health Sciences, University of the State of Mato Grosso (UNEMAT), Cuiabá, Brazil; 3 Faculty of Nursing, Federal University of Mato Grosso (UFMT), Cuiabá, Brazil; 4 Swiss Tropical and Public Health Institute (Swiss TPH), Basel, Switzerland; 5 University of Basel, Basel, Switzerland

**Keywords:** Leprosy, Physical disability, Cure, Progression, Survival

## Abstract

**Background:**

Leprosy can be cured, but physical disability (PD) as a result of the infection can progress in the post-release from treatment phase. This study evaluated the likelihood of, and factors associated with, the progression of the PD grade post-release from treatment among leprosy patients treated in Cáceres-MT, Brazil in the period 2000 to 2017.

**Methods:**

A retrospective cohort study and survival analysis were performed in the hyperendemic municipality of Cáceres in the state of Mato Grosso. The study population consisted of newly diagnosed leprosy patients released from treatment between January 1, 2000 and December 31, 2017. The main outcome was the progression of the PD grade with regard to probability and time; and the evaluated covariates included clinical, operational and demographic variables. The Cox proportional risk model was used to estimate the risk ratio (Hazard Ratios) of the covariates. Both an univariate and a multivariate analysis were implemented, with 95% confidence intervals.

**Results:**

The mean time for progression of the PD grade was 162 months for PB and 151 months for MB leprosy patients. The survival curve showed that 15 years after the release from treatment, the probability of PD grade progression was 35%, with no difference between PB and MB or age groups. Leprosy reactions and registered medical complaints of any kind during treatment were identified as risk factors with Hazard Ratios of 1.6 and 1.8 respectively.

**Conclusions:**

People released from treatment as cured of leprosy are susceptible to worsening of the PD, especially those who have had complications during multi-drug therapy treatment. This indicates that leprosy patients should be periodically monitored, even after the successful completion of multidrug therapy.

## Background

Physical disability (PD) can occur before leprosy diagnosis, during treatment and post-release from treatment [1]. However, although patients may be exposed to risk factors that potentiate the risk of more severe PD [2, 3] they stop being routinely evaluated once the treatment of active leprosy has been completed [4]. Any worsening of the PD of leprosy patients post-release from treatment is worrying, and a lack of comprehensive and systematic follow-up health care can leave cured leprosy patients neglected and vulnerable [3]. Many patients are released from treatment with leprosy reactions and neural impairment [5, 6] but are then excluded from active review of their disease status and adequate management of their conditions [7]. In Brazil, newly diagnosed leprosy patients are systematically recorded and monitored during multidrug therapy (MDT) and are considered cured at the end of treatment according to the paucibacillary (PB) or multibacillary (MB) treatment protocol, depending on the initial diagnosis [7]. In the period following release from treatment, the prevention of PD is based on self-care and patients are instructed to self-refer to health services in case they detect signs and symptoms of neural impairment [7].

The relevance of PD among leprosy patients is widely acknowledged. It is also well known that it can occur post-release from treatment either due to late detection [2, 8], late onset of leprosy reactions [3, 5, 9] or in the absence of qualified and timely assistance [10]. The lack of adherence to recommended self-care for prevention of PD as another contributing factor during treatment [11] and post-discharge has also been documented [12]. Unfortunately, the period post-discharge appears to be of minor concern to health authorities as indicated by the limited space and attention it receives in the guidelines on the recommended activities to prevent PD [7, 13].

According to WHO statistics for 2017, the number of leprosy cases diagnosed with grade 2 PD declined globally. The exception were five countries including Brazil where the figure increased by 10% over the previous year [14]. The Global Leprosy Strategy 2016–2020 has set as one target the reduction of leprosy cases with grade 2 PD to less than 1 per million inhabitants [15]. Of note, the leprosy burden is calculated based on the Disability-Adjusted Life Year (DALY) indicator, that combines mortality and morbidity information to measure the impact of disease on population health [16]. As leprosy is associated with low mortality, PD represents the main burden of disease due to leprosy, but the indicator only covers information collected at diagnosis and during treatment whereas the period post-release from treatment is not included [2, 8].

Considering the thousands of leprosy patients treated and released from treatment annually, and the long-term consequences of PD, the aim of this study was to evaluate the likelihood and factors associated with the progression of the PD grade post-release from treatment among leprosy patients in Cáceres-MT, Brazil in the period 2000 to 2017.

## Methods

### Study population and data sources

All leprosy patients treated and cured at the referral center for leprosy patients and eight urban units of the Primary Health Care System in the city of Cáceres were included in a retrospective cohort study and survival analysis with months as the unit of time. Cáceres is located in the western region of the state of Mato Grosso, the most leprosy-endemic state in Brazil and bordering the states of Mato Grosso do Sul, Goiás and Pará. It also shares an international border with Bolivia. As it is the case throughout Brazil, leprosy care is decentralized to the primary care facilities, with support by leprosy reference centers.

Release from treatment is defined as the end of treatment with MDT [7]. It is recorded as exit by cure in the leprosy monitoring bulletin in the National Information System of Notifiable Diseases (SINAN) [17].

The study population consisted of newly diagnosed leprosy patients who were released from treatment between 1 January 2000 and 31 December 2017. The study included mainly patients living in urban areas, all diagnosed and treated in the municipality and with a record of the assessment of the PD grade at diagnosis and at release from treatment. Exclusion criteria for the study were cases of grade 2 physical disability (G2D) at release from treatment because they already had the highest disability grade, according to the criteria of the Ministry of Health - Brazil [7, 18]. Follow-up losses were due to addresses which could not be verified, death, relocation outside the study area, and refusal to participate in the study.

### Variables

The progression of the PD grade in the post-release from treatment period was defined as main outcome of the study. The term “progression” represents any worsening or advance in the PD and consequently, non-progression represents the maintenance or reduction of the PD grade. The considered covariates were: gender (male and female); educational level (illiterate, elementary school, high school, higher education and missing); age group (6–15, 16–29, 30–59 and 60 and more years); leprosy classification (PB and MB); leprosy reactions during treatment (yes/no); leprosy-related medical complaints during treatment (yes/no).

The addresses registered in the medical records and complementary information by the Community Health Workers and other health workers supported the identification of the current location of the participants. Data were obtained from medical records and a simplified neurological evaluation, and registered in a spreadsheet.

The simplified neurological assessment was performed to determine the current (= time of the study) grade of PD pertaining to the eyes, hands and feet [7]. Thus, the time between release from treatment and the assessment varied between participants. The single assessment was performed in a health facility near the participants’ residence, by trained health workers supported by the researchers with experience in neurological assessment of leprosy patients. The PD grade was classified as follows: grade 0–G0D (no leprosy-related physical disability), grade 1–G1D (decreased strength and/or loss of sensation) and grade 2–G2D (presence of visible disabilities and deformities) [7, 13]. Individuals with open lesions requiring immediate care received assistance at home and, when necessary, were referred for care at the health unit closest to their residence.

The follow-up time was recorded in months and defined as the period between the date of release from MDT treatment and the post-discharge neurological assessment.

### Statistical analysis

The Chi-square test was used to evaluate the proportion difference between covariates. To estimate Hazard Ratios (*HR*) of covariates, in both the univariate and multivariate analysis, the Cox proportional risk model was used, and 95% confidence intervals (95% *CI*) were calculated using the Wald test for each covariate. In the final model the adjusted variables were considered as well as the overall chi-square. Survival curves were constructed using the Kaplan-Meier method to assess the likelihood of leprosy PD progression in the post-release from treatment period. The log-rank test was used to evaluate the differences between the MB and PB groups regarding the delay until progression of the PD grade. Individuals who did not show progression of the PD grade and those not evaluated at follow-up (lost at follow-up) were censored at the end of the study. Statistical analysis was performed using the Statistical Package for the Social Sciences program (SPSS version 22.0; IBM Corp., Armonk, NY, USA).

The study was approved by the Ethics Committee of the Hospital Universitário Júlio Muller (authorization number 555567; 02/26/2014). All participants signed a Consent Form after they had received an oral and written explanation of the study during a visit to their home by a researcher and health workers.

## Results

### Study cohort characteristics

**Fig. 1:**
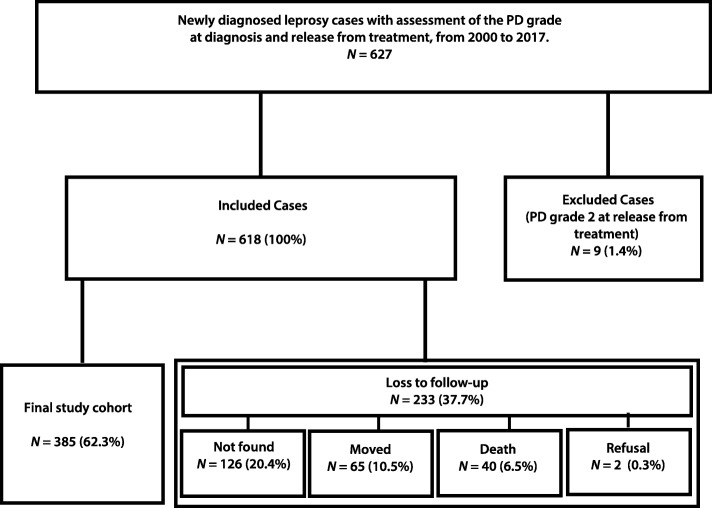
Flowchart of the follow-up of a cohort of leprosy patients released from treatment in Cáceres-MT, Brazil, in the period 2000 to 2017. Potentially eligible were individuals with an assessment of the physical disability grade at diagnosis and release from MDT treatment who were still living in Cáceres at the time of the study

Among the 618 potential participants initially considered eligible for inclusion in the study, 62.3% (*n* = 385) could be located and agreed to the assessment of the PD grade in the frame of the study. Unavailable potential participants were not located at the recorded address (20.4%), moved out of the municipality (10.5%), had died (6.5%) or refused to participate in the study (0.3%; Fig. 1)**.**

No statistically significant differences with regard to gender, age group or operational leprosy classification were observed between the final study cohort and the patients lost to follow-up (Table 1).

**Table 1:** Comparison of key characteristics between the final study cohort of leprosy patients and potential study participants from Cáceres - MT, Brazil (index patients released from treatment in 2000 to 2017)

Variables	Final study cohort ***n*** (%)	Lost to follow-up ***n*** (%)	***χ***^**2**^	***P***-value
**Gender**				
Male	200 (59.3)	137 (40.7)	2.747	0.097
Female	185 (65.8)	96 (34.2)	1	-
**Age group (years)**				
6–15	18 (51.4)	17 (48.6)	0.396	0.528
16–29	76 (58.0)	55 (42.0)	0.487	0.484
30–59	234 (66.3)	119 (33.7)	3.088	0.078
60 +	28 (28.3)	71 (71.4)	1	-
**Operational classification**				
Paucibacillary	252 (60.7)	163 (39.3)	1	-
Multibacillary	133 (65.5)	70 (34.5)	1.334	0.248

-: Not applicable.

### Risk of physical disability grade progression

The Cox proportional risk model indicated no significant difference in the risk of progression of the PD grade post-release from treatment between age groups and operational leprosy classifications. Leprosy reactions during the treatment period represented a 1.6 times greater risk of PD progression (*HR*: 1.6; 95% *CI*: 1.1–2.4). A record of leprosy-related complaints during treatment was associated with almost twice the risk of progression of the PD post-release from treatment (*HR*: 1.8; 95% *CI*: 1.3–2.4; Table 2).

**Table 2:** Hazard Ratio (*HR*) and 95% confidence intervals (*CI*) for the progression of the physical disability grade among leprosy patients in the post-release from treatment period, Cáceres - MT, Brazil (index patients released from treatment in 2000 to 2017)

Variables	Progression (***n***)	Crude ***HR*** (95% ***CI***)	***P***-value*	Adjusted ***HR*** (95% ***CI***)^**a**^	***P***-value*
**Gender**					
Male	103	1.06 (0.79–1.41)	0.689	**-**	**-**
Female	85	1		**-**	
**Age group (years)**					
6–15	9	1		-	-
16–29	27	0.78 (0.36–1.66)	0.525	-	
30–59	123	1.53 (0.78–3.02)	0.213	-	
60 or older	29	1.23 (0.58–2.61)	0.579	-	
**Operational classification**-					
Paucibacillary	123	1		-	-
Multibacillary	65	0.82 (0.60–1.11)	0.198	-	
**Leprosy reaction during treatment**					
Yes	71	2.09 (1.55–2.82)	0.001	1.65 (1.13–2.40)	0.008
No	116	1		1	
**Complaints during treatment**					
Yes	82	2.01 (1.49–2.70)	0.001	1.76 (1.29–2.40)	0.001
No	102	1		1	

^a^ Variables adjusted by leprosy reaction during treatment, complaints during treatment

^*^P-value according to Wald test

-: Not applicable; *HR* - Hazard ratio; *CI* - Confidence interval.

**Fig. 2:**
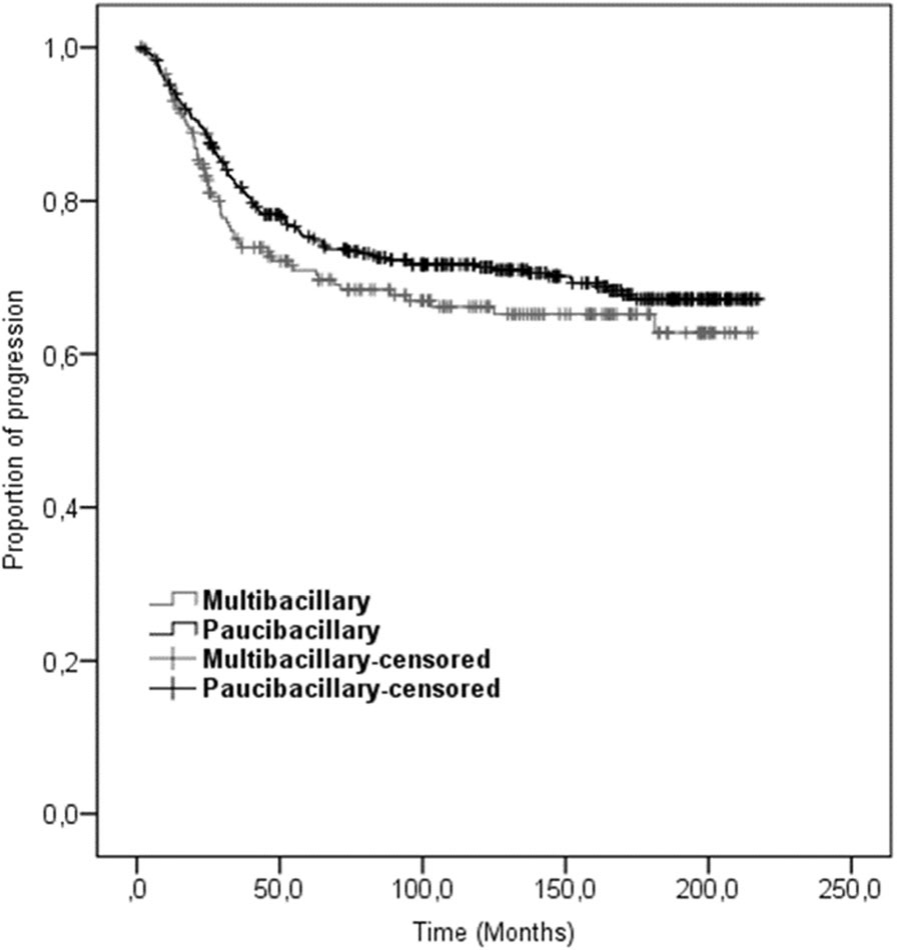
Survival curve for PD grade progression post-release from MDT treatment for MB and PB leprosy patients released from treatment in 2000–2017; Cáceres – MT, Brazil

The mean time for progression of the PD grade was 162 months for PB and 151 months for MB. The survival curve shows that after release from MDT treatment the risk of PD grade progression was similar for patients with PB and MB leprosy (*P*-value > 0.05; Fig. 2).

The mean time until progression of the PD grade was 130 months for patients with documented leprosy reactions during treatment. For individuals with recorded complaints during treatment the average time of progression of the PD grade was 139 months.

## Discussion

Our study shows that the cumulative probability of progression (worsening) of the PD grade of people treated and bacteriologically cured of leprosy increases with time. After 10 years this probability was 30%, and after 15 years it was 35% in a cohort of patients from Mato Grosso, Brazil. The risk of disability progression is associated with leprosy reactions and the presence of triggers for complaints during the MDT treatment phase. It is possible that some individuals rated with more severe PD at follow-up compared to the moment when they were released from treatment developed this condition before or during treatment, but it went unrecorded. However, particularly G2D (more severe) is characterized by visible deformities, generally precluding classification errors.

Peripheral neuropathies can be irreversible and cause emotional, social and economic problems to those affected [19]. Preventing disability is one of the priorities of the Global Leprosy Strategy. The emphasis of the WHO strategy is early detection to prevent cases with G2D at the time of diagnosis [15]. The relevance of early detection as an approach to reduce the risk of developing disabilities is undisputed [20]. Studies conducted in the post-release from treatment period showed that early diagnosis is associated with reduced risk of PD [2, 8, 21]. On the other hand, any delay in starting treatment increases the risk of PD developing in the post-release period [8]. Descriptive studies conducted in India and Nigeria [6, 22], in Brazil [3, 19, 23] and specifically in Mato Grosso [24] confirm our findings of considerable PD developing after treatment completion. For this reason, disability prevention measures including regular medical review of the former patients are recommended to continue after the completion of the MDT treatment. For this, primary care would need to be restructured to ensure the provision of standardized and quality services post-release from treatment [7, 25].

Our findings are also corroborated by observations on the cohort of MB leprosy patients treated at the Oswaldo Cruz Foundation (FIOCRUZ), Rio de Janeiro. A 40% probability of progression of PD 10 years after MDT completion has been documented for this group. The main difference between the two cohorts is that the present study followed up MB and PB patients in a hyperendemic area of Brazil who had been treated in primary care while the study by Sales et al. [2] focused on the follow-up of patients seen by referral service. This patient population tends to be more severely affected when compared to those treated by the primary care services. Indeed, Rio de Janeiro is considered low endemic for leprosy; however, the average patients have more severe complications when compared to their peers from Mato Grosso. From 2012 to 2016, the average new case detection rate (NCDR) in Rio de Janeiro was 7.2 cases per 100 000 inhabitants; in the same period, the rate of patients with G2D was 7.4 cases per 1 million inhabitants. In Mato Grosso, the NCDR was 88.9 per 100 000 inhabitants and the rate of patients with G2D was 446.6 cases per 1million inhabitants over the same period [26].

It is noteworthy that the multivariate analysis showed no significant difference between PB and MB cases with regard to the rate of PD progression. Of note, the classification of leprosy patients had to be taken at face value and could not be independently verified. Operationally, this means that all leprosy patients have a potential to develop PD post treatment completion and need to be considered in efforts to prevent leprosy sequelae. It is thus important to launch disability prevention activities for all patients, regardless of the clinical form and the treatment status.

Leprosy reactions are the main risk factor for the development of PD during [5, 22] and after treatment [6]. Our findings show that the occurrence of leprosy reaction episodes during MDT treatment increases by 96% the risk that the disability grade progresses to a more serious stage post-release from treatment. This corroborates findings by others [3, 8, 27]. In contrast, a study of treated MB leprosy patients revealed no association between leprosy reactions and the development of new disabilities, possibly suggesting that the worsening of the PD grade in the post-release period depends on the clinical management of cases during treatment [2].

Our findings show no association between age and the worsening of the PD grade in the post-release from treatment period, in accordance with other studies [2, 8]. Age may reflect late diagnosis and delayed treatment, which are the main causes of PD in adults [8] but apparently does not influence in itself the worsening of PD after treatment.

The development of PD can result in reduced physical capacity, the latter being essential for many professional activities [24]. It can thus render individuals more susceptible to unemployment and socially vulnerable [4, 28]. Being unemployed can interfere with people’s quality of life, compromise the self-support and financial maintenance of the family, causing social, psychological and economic problems [29]. Studies in India [30] and Brazil [3, 10] have described these relationships in detail.

Adherence to regular practice of self-care is low in former leprosy patients due to stigma, lack of knowledge about leprosy, inability to perform activities and lack of commitment to health [9]. The participation of cured patients in a self-care group contributes significantly to the acquisition of knowledge and improves self-care practices [31]. In Mato Grosso, there are currently no self-care groups in a majority of municipalities. After release from MDT treatment, people are no longer regularly seen by the health services and are advised to only return when identifying a neural disorder [7]. This recommendation may not be appropriate considering the socioeconomic profile of people who develop PD due to leprosy. Generally, the affected population has limited education and income [3, 19, 32], factors that may interfere with the understanding of the concept of self-care, with self-observation and with treatment seeking behavior [3].

Whereas reducing PD at the time of diagnosis is currently a priority for WHO and many countries [15], it is necessary to reorganize leprosy programs to offer care for all individuals affected by the disease, including those released from MDT treatment. The post-release care process should be standardized and the care structure available in the primary care network improved in view of the continuing demand for these services [3, 4, 23]. Of note, such services must complement self-care and -check by those affected by the disease but released from treatment to jointly contribute to the prevention of PD [11].

The Brazilian guidelines on leprosy patient care as well the training for leprosy prevention, diagnosis and treatment suggest a simplified neurological assessment. For this study, the PD grade assigned by the health services at the time of the assessment was used. The accuracy of these assessments is unknown. Also, changes in the PD not warranting a re-classification of the PD grade were not considered in the study.

## Conclusion

The risk of worsening physical disabilities due to leprosy over a 15 year follow-up period is high. Indeed, more than one third of the individuals treated and registered as cured by health services in a hyperendemic area in Brazil showed clear signs of PD worsening. Especially people released from treatment as cured but who have had complications during treatment with MDT are at risk. Leprosy patients should be regularly examined for signs of disability even after the completion of MDT.

## Data Availability

From first author.

## Abbreviations

G2D: Grade 2 disability
*HR*: Hazard ratio
*CI*: Confidence interval
PB: Paucibacillary
MB: Multibacillary
MDT: Multidrug therapy
NCDR: New cases detection rate
PD: Physical disability
WHO: World Health Organization
